# Retinal Nerve Fiber Layer Changes after Intraocular Silicone Oil Tamponade in Rhegmatogenous Retinal Detachment

**DOI:** 10.3390/vision7010013

**Published:** 2023-02-21

**Authors:** Fitri Annur Chikmah, Andi Muhammad Ichsan, Itzar Chaidir Islam, Joko Hendarto, Habibah Setyawati Muhiddin, Budu

**Affiliations:** 1 Ophthalmology Department, Faculty of Medicine, Hasanuddin University, Makassar 90245, Indonesia; 2 Hasanuddin University Hospital, Makassar 90245, Indonesia; 3 JEC Orbita, Makassar 90222, Indonesia; 4 Public Health Medicine Department, Faculty of Medicine, Hasanuddin University, Makassar 90245, Indonesia

**Keywords:** silicone oil tamponade, rhegmatogenous retinal detachment, retinal nerve fiber layer, central macular thickness

## Abstract

Rhegmatogenous retinal detachment (RRD) is a serious and emergency condition that may cause visual disturbance. Treatment includes pars plana vitrectomy with a tamponade such as intraocular gas or silicone oil (SO). In many countries, silicone oil is still favorable compared to intraocular gases as tamponade for reattachment of retinal detachment surgery. The application provides a higher anatomical success rate, especially in cases of proliferative vitreoretinopathy (PVR) that were previously considered untreatable. Objective assessment of the retinal nerve fiber layer (RNFL) using optical coherence tomography (OCT) in the eye with silicone oil tamponade is a challenge because of the limitations and difficulties in taking images. This study aims to assess the RNFL thickness changes in rhegmatogenous retinal detachment patients using SO tamponade and its subsequent removal conducted on a total of 35 post-operative RRD patients. Central macular and RNFL thickness, as well as best-corrected visual acuity (BCVA), were recorded at the time of tamponade and after the removal of the SO at 1, 4, and 8 weeks, respectively. The results showed that the changes in RNFL thickness significantly decreased in the group of ≤6 months, especially in the superior and temporal quadrants, and BCVA increased after SO removal (*p* < 0.05). Central macular thickness was significant (*p* < 0.001) at the end of the visit. Improved visual acuity is associated with decreased RNFL and central macular thickness after SO removal.

## 1. Introduction

Rhegmatogenous retinal detachment (RRD) is the separation of the neurosensory layer retina from the retinal pigment epithelium (RPE) with a full-thicknesss break in the retina. In most cases, these breaks are brought about by vitreous traction on the retina, which also makes it possible for fluid to accumulate in the subretinal region [1]. This pathologic condition is devastating and requires immediate treatment as it may result in vision loss. The number of cases is a prevalence of 1 in 10,000 cases per year [2]. Age, gender, history of cataract surgery, and myopic status are all variables that might increase the likelihood of developing rhegmatogenous retinal detachment. There is an increased risk of RRD in myopic patients by a factor of ten for every three dioptres. In Asia, the rate of high myopia among school-aged children is as high as 80% [3]. The risk of RRD varies not just by myopic status—White and Asian males have a relatively higher risk than other groups [4].

The treatment of RRD includes surgery of pneumatic retinopexy (PnR), scleral buckling (SB), and pars plana vitrectomy (PPV). Pneumatic retinopexy is a non-incisional, minimally invasive surgical surgery initially reported by Rosengren in 1938 [5]. It is used to cure rhegmatogenous retinal detachment with the location of superior breaks. In PnR, the fundamental surgical processes include retinopexy of the retinal break by using cryotherapy or laser photocoagulation, intraocular gas injection either before or after retinopexy, and the maintenance of an appropriate head position for the required amount of time following surgery [6,7].

Scleral buckling is a surgical procedure that repairs retinal breaks and reduces vitreous stress on retinal tears. Since the 1950s, SB has been used as either the primary or secondary treatment for RRD repair. This approach was inspired by Jules Gonin’s [8], and until now, SB is still the top choice in the treatment of phakic eyes with localized RRD accompanied by small anterior holes or retinal dialysis, especially when the signs of proliferative vitreoretinopathy (PVR) are not present. The buckle creates a depression in the sclera, to reattach the retinal separation of the neurosensory retinal (NSR) layer to the retinal pigment epithelium (RPE). The surgery is based on two key principles: the closing of retinal tears and the creation of a lasting chorioretinal adhesion [9]. Both of these principles are essential to the success of the operation. It has been shown that scleral buckling provides better morphological and functional results in phakic eyes when compared to vitrectomy when the separation is simple or relatively less complicated [10,11].

In some circumstances, RRD are associated with vitreous opacities that obscure the retinal view, giant retinal breaks, posterior retinal breaks that cannot be easily reached by buckling or any related condition with vitreoretinal traction that cannot be relieved by SB [12]. In cases of retinal detachment requiring PPV, tamponade agents such as intraocular gases or silicone oils (SO) are used to restore intraocular volume and apply surface tension to the entire detached retinal surface [13]. In contrast to PnR, which makes use of intraocular gases that are not diluted and expand, tamponade in PPV is typically achieved by completely filling the vitreous cavity with non-expanding gases that have been diluted in the air at isovolumetric concentrations (for example, 20% SF6 or 14% C3F8). This is done in order to prevent the vitreous cavity from being displaced [14,15].

The application of SO provides a higher anatomical success rate, especially in cases of PVR that were previously considered untreatable [16,17]. SO must displace retinal aqueous humor to work as an internal tamponade. This function depends on four physical parameters, including specific gravitation, buoyancy, interfacial tension, and a viscosity [18]. Silicone oil floats in the vitreous cavity because the specific gravity is 0.97; its bubbles’ surface tension may change after injection into the eye. Higher viscosity silicone oils may emulsify less. In the vitreous cavity, buoyancy and gravity operate on an intraocular tamponade agent that presses against the retina as a downward force. Moreover, interfacial tension is the interaction between two immiscible chemicals, such silicone oil and aqueous humor. Current silicone oils have viscosities ranging from one thousand (MW 37 kDa) to five thousand cSt (MW 65 kDa) [18,19].

A study of SO tamponade in rabbit eyes showed a significant reduction in myelinated optic nerve fibers. Human and animal studies report silicone oil migration to ocular tissues, including the optic nerve and macrophage-mediated inflammatory responses. The objective assessment of the retinal nerve fiber layer (RNFL) in the eyes with intraocular SO tamponade is difficult in taking image. Optical coherence tomography (OCT) is a non-contact and non-invasive technology used to describe and monitor retinal layers and optic nerve morphology. It can detect retinal nerve tissue loss by quantitatively measuring RNFL thickness at high resolution [20,21,22,23]. Meanwhile, recent advances in vitreoretinal surgery have improved surgical outcomes [24]. Various factors, including the height of the macular detachment and outer retinal subfoveal changes, have been evaluated for visual acuity outcomes in RRD [25].

This study aims to assess the retinal nerve fiber layer thickness changes, intraocular pressure and central macular thickness and their correlation with the best corrected visual acuity outcomes in rhegmatogenous retinal detachment patients using silicone oil tamponade and its subsequent removal.

## 2. Materials and Methods

This study was a prospective cohort study conducted at the Hasanuddin University Hospital and JEC-ORBITA eye clinic, Makassar, Indonesia, to evaluate the changes in retinal nerve fiber layer thickness and central macular thickness in patients of rhegmatogenous retinal detachment when using an intraocular tamponade of silicone oil and after its removal.

A total of 35 patients fulfilled the inclusion criteria and underwent pars plana vitrectomy followed by silicone oil as the intraocular tamponade. The range in patient age was 15–60 years old; the patients that showed a willingness to participate in the study signed the informed consent. Meanwhile, the exclusion criteria were the presence of macular abnormalities, such as an epiretinal membrane (ERM), macular hole, and all cases requiring internal limiting membrane (ILM) or ERM peeling, glaucomatous optic neuropathy, and non-cooperative patients. Others with a history of ocular trauma and retinal vascular disease were also excluded. Patients are declared dropouts when they did not follow up according to the time schedule and experienced retinal redetachment after the removal of silicone oil.

The silicone oils used were SO 1300 and 5000 cSt, with the duration of intraocular tamponade ranging from 3 to 12 months. Silicone oil removal was performed when complete retinal attachment status was achieved, or there were any signs of silicone oil emulsification. All patients who fulfilled the inclusion criteria were examined for visual acuity, anterior segment of the eye, intraocular pressure, indirect fundoscopy, and OCT (Heidelberg engineering, HRA OCT Spectralis^®^*)* for the examination of RNFL and CMT using three circular scans with a diameter of 3.4 mm for each eye, as well as the macula. This examination was carried out serially before (group 1) and after SO removal at 1 week (group 2), 4 weeks (group 3), and 8 weeks (group 4). All results were recorded and analyzed using paired *t*-tests and repeated ANOVA followed by the post-hoc Bonferroni test, sig. *p* < 0.05.

## 3. Results

**Figure 1:**
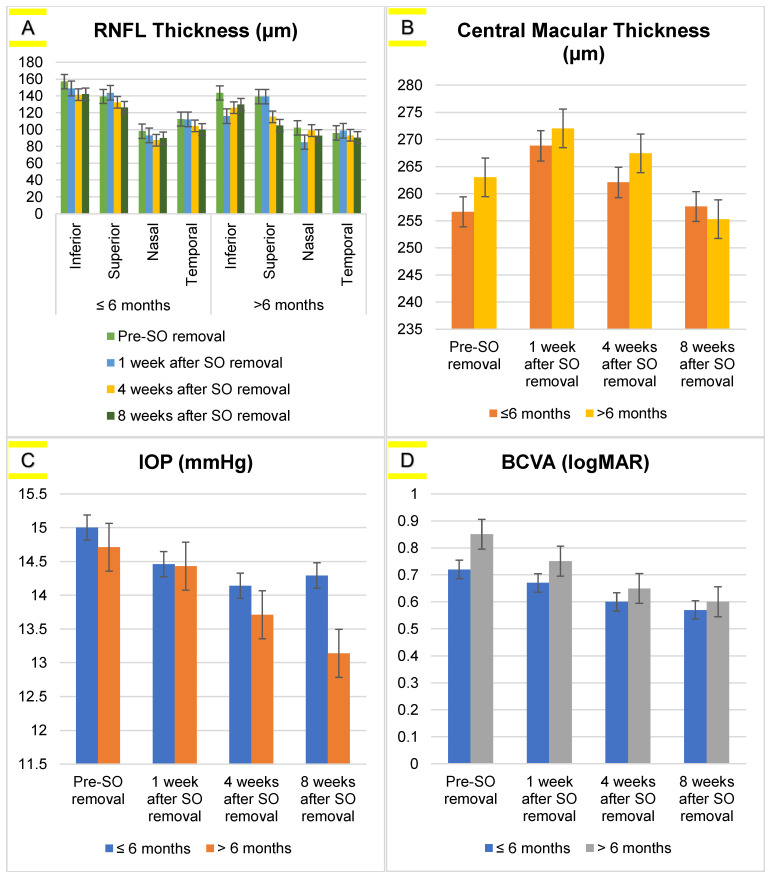
Average values of (**A**) RNFL thickness, (**B**) CMT, (**C**) IOP, and (**D**) BCVA based on the duration of use SO on measurement time of pre- and post-silicone-oil removal in rhegmatogenous retinal detachment patients.

**Figure 2:**
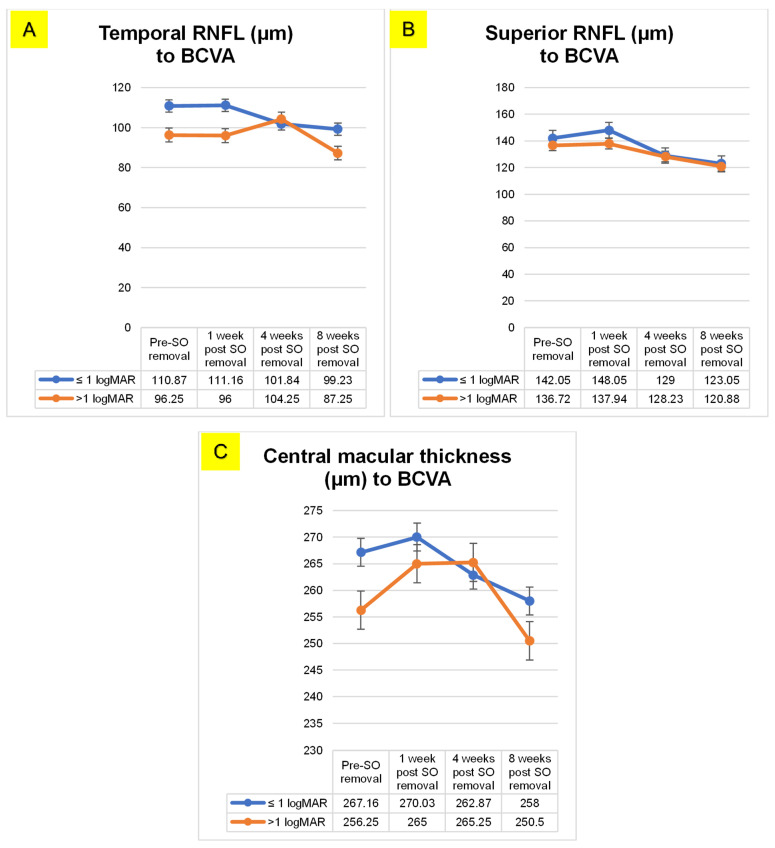
(**A**) Temporal RNFL thickness, (**B**) Superior RNFL thickness, and (**C**) Central Macular thickness of BCVA in rhegmatogenous retinal detachment patients pre- and post SO removal.

The mean differences in retinal nerve fiber layer thickness, central macular thickness, intraocular pressure, and best-corrected visual acuity before and after SO removal are shown in Table 1. In Figure 1, the data was divided based on the intraocular tamponade SO duration (≤6 months and >6 months). Statistical analysis found there were significant differences between the RNFL thickness ≤6 months in the superior (*p* < 0.001) and temporal (*p* < 0.001) areas, CMT ≤ 6 months (*p* < 0.001), and the BCVA measurements ≤ 6 and >6 months (*p* < 0.001). Therefore, a post-hoc analysis was performed on RNFL thickness, CMT, and BCVA based on the duration of silicone oil as displayed in Table 2. Moreover, the correlation of the significant value of RNFL (superior and temporal) thickness and CMT with BCVA is shown in Figure 2.

Based on Table 1, it can be seen that RNFL thicknesses was significantly decreased at 4 and 8 weeks after SO removal compared to pre SO removal (*p* < 0.05). Similar results were found in CMT, wherein the central macular thickness significantly decreased post SO removal (*p* < 0.001). The IOP did not show any significant difference between pre- and post SO removal (*p* > 0.05). Meanwhile, BCVA showed an increased value after SO removal (*p* < 0.001).

Table 2 shows post-hoc analysis of best corrective visual acuity, central macular thickness, and retinal nerve fiber layer thickness, there are significant difference among groups, and group 4 (8 weeks post SO removal) is the most significant improvement in all variables (*p* < 0.05).

Figure 2 shows the relationship between retinal nerve fiber layer thickness and CMT with BCVA. In Figure 2A,B it can be seen that there is a decrease in the thickness of the retinal nerve fiber layer both on the superior and temporal sides before and after SO removal. Similar results were also shown by the comparison of CMT and BCVA (2C) that macular thickness decreased with the duration of follow-up.

## 4. Discussion

In this study, the viscosity of silicone oil that was mostly used was SO 1300 cSt for the primary reattachment surgery and 5000 cSt for the redetachment patients. It is similar to a study by Soheilian et al. (2006), who reported that the use of SO 5000 cSt was associated with a high incidence of retinal redetachment after SO removal [26]. A study by Kartasasmita et al. (2017) found that SO 1000 emulsified more than SO 5000 [27]. A retrospective study by Scott et al. (2006) on 325 eyes with complex retinal detachment with anatomic success rates and visual acuity had no significant differences between SO 1300 and 5000 cSt [28].

In this study, the mean BCVA before silicone oil removal was 0.75 LogMAR, but afterward, it improved to 0.69, 0.61, and 0.58 at 1, 4, and 8 weeks post SO removal, respectively. Similar results were found by Selim et al. (2019), who assessed BCVA before and 8 weeks after removal; the BCVA was 0.05 dec and 0.05–0.8 dec, consecutively [29]. A study by Nassar et al. (2019) also reported that 6 months or >6 months of SO application affected BCVA. In a recent macula-on retinal detachment study, higher IOP during SO endotamponade was the biggest risk factor for vision loss [30]. Abu Al Naga et al. (2019) and Ghada et al. (2019) reported that BCVA improves by 1.06–2.1-fold 4 weeks after removal (*p* < 0.05), and the mean IOP before and after 4 weeks of removal were 20.18 mmHg and 14.18 mmHg (*p* = 0.025) [31].

Our study found that IOP was not similar with Nassar et al. (2019), increased IOP may damage the fovea through mechanical stress and can cause loss of the outer nuclear layer cell bodies. Increased IOP may mechanically stress the fovea, causing outer nuclear layer cell body loss. Thus, this drop in IOP may have improved retinal sensitivity. In a recent macula-on retinal detachment study, higher IOP during SO endotamponade was the biggest risk factor for vision loss significantly different at pre- and post SO removal (*p* = 0.08). This result is similar to the study by Brănişteanu et al. (2017), who reported a decrease in IOP post SO removal [32].

Saleh et al. (2020) reported a different result in which IOP significantly increased from the baseline value when using SO endotamponade, from 15 ± 5 mmHg to 20 ± 11 mmHg (*p* < 0.001). However, after removal, it significantly reduced to 15 ± 6 mmHg at the last visit with *p* < 0.001 [33]. Several reports also showed that the first sign of SO emulsification can be found within the first 3 months post-operatively, or even 4 weeks after endotamponade. Due to a large number of cases of SO emulsification within 1 year, the consensus recommended that removal must be carried out within this time interval [32,33]. The mean IOP for all age groups and durations of SO application did not affect pre removal measurements or follow up. According to Issa et al. (2020), who studied post SO removal complications, IOP pre removal was 15.7 ± 5.1 mmHg and decreased to 15.0 ± 5.8 mmHg at the second month of follow up. Jawad et al. (2016) observed changes in IOP during SO tamponade and after removal. The mean of IOP measurements in pre SO removal was 27.35 ± 9.20 mmHg, but it decreased to 16.10 ± 14 mmHg after 6 months [34,35,36].

In this study, the mean of CMT was 265.91 ± 20.01 µm. In the first week post SO removal, it was 269.46 ± 18.52 µm, then gradually decreased to 263.14 ± 22.14 µm and 257.16 ± 22.17 µm after 4 and 8 weeks. Dugyu et al. (2021) reported there was an increase in CMT values after 1 month SO removal. This is presumably associated with inflammation and the incidence of central macular edema (CME). The inflammatory response to SO tends to continue until post SO removal. The CMT area was reduced alongside the decrease in inflammatory response, which improved visual acuity [37].

Because of the wide disparity in CMT values depending on several factors such as age, gender, and ethnicity, it is possible to get more consistent findings by comparing the CMT values of both eyes belonging to the same person [38]. Following tamponade with silicone oil, Bae et al. (2019) found that the structure of the participants’ macular tissue was altered in 46 patients, epiretinal membrane (26.1% of cases), cystoid macular edema (19.6% of cases), and a decrease in the thickness of the central macular area were the changes that occurred in the retinal structure. Once the silicone oil was removed, these alterations were able to be recovered [39].

A recent study conducted by Rabina et al. (2020) reported that 41 patients showed a temporary decrease in retinal thickness, particularly in the inner retinal layers. However, after the silicone oil was removed from their eyes, these patients’ retinas regained the thickness levels of a healthy structure [40]. Another study that included 10 people found that a tamponade of silicone oil caused the fovea to become flatter. Following the removal of the silicone oil, the phenomenon reverted, and the fovea reclaimed the thickness it had before the operation [38].

The thickness of the subfoveal choroidal layer and the retinal layer both reduced noticeably as a result of the SO tamponade [41]. According to the findings of the study conducted by Kheir WJ et al. (2018), CMT levels dropped when the SO tamponade was applied, but they increased when the SO was withdrawn. Nevertheless, these changes did not reach the level of statistical significance (*p* = 0.44) [42]. In addition, the inner retinal layers were shown to be much thinner in the presence of SO tamponade in comparison to healthy eyes in two separate tests that were carried out by Purtskhvanidze et al. and Caramoy et al. [43,44].

During tamponade, the RNFL thickness was measured and continuously evaluated until 8 weeks after SO removal. Eight weeks after the removal, the RNFL thickened in the nasal quadrant from 98.97 ± 34.50 µm to 90.40 ± 31.43 µm, in the temporal area 109.20 ± 44.92 µm to 97.86 ± 31.23 µm, in the superior area 139.31 ± 34.71 µm to 121.94 ± 25.47µm, and in the inferior area 154.31 ± 44.05 µm to 139.69 ± 36.38 µm. In this study, superior and temporal nerve fiber layer thickness were significantly decreased at 8 weeks after SO removal (*p* < 0.001). Takkar et al. (2018) reported similar results, with the temporal quadrant having the lowest mean RNFL thickness after removal at 51 µm, followed by nasal 65 µm, superior 85 µm, and inferior 94 µm. The temporal and inferior quadrants increased before and after removal, at 26% and 21%, respectively [45]. Another study found that RNFL thickness increased in all quadrants after SO removal compared to pre removal. In the area of inferior and superior, the RNFL thickness decreased after 2 years of SO removal [46]. Lee et al. (2012) described RNFL thickness in RRD patients with retinal detachment. At 6,12, and 24 months after endotamponade, values were 113.9 ± 13.5 µm, 108.8 ± 15.1 µm, and 104.5 ± 14.2 µm. The results showed decreased value during the follow-up period, but there were no post removal measurements. SO tamponade can affect the retinal structure, and several hypotheses have been proposed [24]. Takkar et al. (2018) stated that potassium accumulation and nerve degeneration cause retinal thinning, while Sebastian et al. (2003) stated that it may be caused by mechanical stress. SO toxicity and dehydration are also hypothesized as potential retinal thinning mechanisms [36,45].

Raczynska et al. (2018) reported the effects of silicone oil on ganglion cell complex (GCC) and compared it to other endotamponades, such as sulfur hexafluoride gas (SF6) and perfluoropropane gas (C3F8). Spectral-domain (SD) OCT showed a significant reduction in average GCC thickness in practically all sectors in the silicone oil endotamponade group at all follow-up visits, despite no visual complaints or scotomas. After surgery, macula status did not change the mean of GCC [47].

Silicone oil intraocular tamponades are safe and widely used. Several studies recommended that SD-OCT patients with silicone oil tamponade should be carefully monitored to identify early changes in the inner retinal layer thickness [48,49]. During SO application and its removal, BCVA correlated with central macular thickness and RNFL thickness. In RRD patients with pre removal, BCVA ≤ 1 and >1 LogMAR, temporal RNFL thickness was 110.87 µm and 96.25 µm, respectively. The value dropped to 99.23 µm for ≤1 LogMAR and 87.25 µm for >1 LogMAR in 8 weeks after SO removal.

Temporal RNFL thickness changes correspond to the macula, which means that the most active sites are more susceptible to retinal detachment injury and microenvironmental changes. The foveola relies on choroidal blood vessels for oxygen and nutrition. Macular detachment and antegrade neuronal degeneration can affect the second and third neurons in the relay [45]. Rabina et al. (2020) reported a transient reduction in central macular thickness. SO thins the retinal component without affecting BCVA, because the mechanical only affects the inner retinal layer and does not permanently damage the photoreceptors, visual acuity is minimally affected [40]. Doslak (1988) stated the electroretinogram (ERG) declined rapidly in silicone oil-filled eyes, the ERG (with a functional retina) was severely reduced to 15% of normal, and even with the most extreme variations of the other parameters, there was still a reduction (60%) of the ERG [50]. Christou et al. (2022) reported that the amplitudes of the a- and b-waves were significantly higher after SO removal than those before SO removal, which means the photoreceptors should have recovered after the silicone oil was removed [51].

## 5. Conclusions

There were statistically significant decreases in retinal nerve fiber layer thicknesses and central macular thicknesses in postoperative rhegmatogenous retinal detachment patients after silicone oil removal, particularly in the superior and temporal quadrants. This result may correlate with corrections and improvement in visual acuity.

## Figures and Tables

**Table 1:** The mean differences in best corrected visual acuity, intraocular pressure, central macular thickness, and retinal nerve fiber layer thickness between pre- and post-silicone-oil removal.

Variables	Measurement Time				***p*** **-Value**
	Pre SO Removal	1 wk Post SO Removal	4 wk Post SO Removal	8 wk Post SO Removal	
RNFL (µm)					
Inferior	154.31 ± 44.05	142.23 ± 38.46	138.34 ± 35.66	139.69 ± 36.38	0.17
Superior	139.31 ± 34.71	142.86 ± 42.86	128.91 ± 27.16	121.94 ± 25.47	<0.001 *
Nasal	98.97 ± 34.50	91.37 ± 28.54	89.77 ± 32.79	90.40 ± 31.43	0.34
Temporal	109.20 ± 44.92	109.43 ± 42.85	102.11 ± 31.79	97.86 ± 31.23	0.02 *
CMT (µm)	265.91 ± 20.01	269.46 ± 18.52	263.14 ± 22.14	257.14 ± 22.17	<0.001 *
IOP (mmHg)	14.94 ± 2.74	14.46 ± 2.72	14.06 ± 2.51	14.06 ± 2.87	0.08
BCVA (LogMAR)	0.75 ± 0.33	0.69 ± 0.29	0.61 ± 0.29	0.58 ± 0.27	<0.001 *

Description: IOP—Intraocular pressure; BCVA—Best corrected visual acuity; * sig., *p* < 0.05. SO—Silicone oil; OCT—Optical coherence tomography, LogMAR—Logarithm of the minimum angle of resolution; RNFL—Retinal nerve fiber layer; CMT—Central macular thickness.

**Table 2:** Post-hoc analysis on best corrected visual acuity, central macular thickness, and retinal nerve fiber layer thickness based on the duration of silicone oil, and central macular thickness on best corrected visual acuity in rhegmatogenous retinal detachment patients.

Variables	Group		Mean Difference	*p*-Value	95% CI	
					Lower	Upper
BCVA ≤ 6 months group	1	2	0.04	0.57	−0.03	0.12
		3	0.11	<0.001 *	0.04	0.19
		4	0.14	<0.001 *	0.06	0.23
	2	3	0.07	0.04	0.00	0.14
		4	0.10	<0.001 *	0.02	0.17
	3	4	0.02	0.37	−0.01	0.07
BCVA > 6 months group	1	2	0.10	0.90	−0.13	0.34
		3	0.19	0.01 *	0.04	0.35
		4	0.25	0.07	−0.02	0.52
	2	3	0.09	0.39	−0.06	0.25
		4	0.14	0.08	−0.01	0.31
	3	4	0.05	1.00	−0.11	0.21
CMT > 6 months group	1	2	−2.17	1.00	−14.63	10.27
		3	4.57	1.00	−9.25	18.39
		4	9.03	0.43	−4.70	22.77
	2	3	6.75	0.15	−1.40	14.90
		4	11.21	0.04 *	0.26	22.16
	3	4	4.46	0.57	−2.91	11.84
RNFL Superior < 6 months group	1	2	8.25	0.06	−0.53	17.03
		3	15.57	0.04 *	0.76	30.38
		4	14.85	0.02 *	2.00	27.70
	2	3	7.32	0.16	−3.13	17.78
		4	6.60	0.19	−3.50	16.72
	3	4	−0.71	0.87	−9.56	8.13
RNFL Temporal < 6 months group	1	2	−4.42	1.00	−13.53	4.67
		3	7.00	1.00	−7.54	21.54
		4	13.17	0.05 *	−0.30	26.65
	2	3	11.42	0.19	−2.96	25.82
		4	17.60	0.01 *	3.06	31.15
	3	4	6.17	0.03 *	0.25	12.10

Group 1: Pre SO removal; Group 2: 1 week post SO removal; Group 3: 4 weeks post SO removal; Group 4: 8 weeks post SO removal. Post-hoc test (Bonferroni), * sig., *p* < 0.05.

## Data Availability

The data presented in this study are available on request from the corresponding author.
